# Characterization and internalization of small extracellular vesicles released by human primary macrophages derived from circulating monocytes

**DOI:** 10.1371/journal.pone.0237795

**Published:** 2020-08-24

**Authors:** Luis A. Arteaga-Blanco, Andrés Mojoli, Robson Q. Monteiro, Vanessa Sandim, Rubem F. S. Menna-Barreto, Filipe Santos Pereira-Dutra, Patrícia T. Bozza, Rafael de Oliveira Resende, Dumith Chequer Bou-Habib

**Affiliations:** 1 Laboratory on Thymus Research, Oswaldo Cruz Institute/Fiocruz, Rio de Janeiro, Brazil; 2 Institute of Medical Biochemistry Leopoldo de Meis, Federal University of Rio de Janeiro, Rio de Janeiro, Brazil; 3 Laboratory of Cellular Biology, Oswaldo Cruz Institute/Fiocruz, Rio de Janeiro, Brazil; 4 Laboratory of Immunopharmacology, Oswaldo Cruz Institute/Fiocruz, Rio de Janeiro, Brazil; 5 National Institute of Science and Technology on Neuroimmunomodulation, Rio de Janeiro, Brazil; VIT University, INDIA

## Abstract

Extracellular vesicles (EVs) are small membrane-limited structures derived from outward budding of the plasma membrane or endosomal system that participate in cellular communication processes through the transport of bioactive molecules to recipient cells. To date, there are no published methodological works showing step-by-step the isolation, characterization and internalization of small EVs secreted by human primary macrophages derived from circulating monocytes (MDM-derived sEVs). Thus, here we aimed to provide an alternative protocol based on differential ultracentrifugation (dUC) to describe small EVs (sEVs) from these cells. Monocyte-derived macrophages were cultured in EV-free medium during 24, 48 or 72 h and, then, EVs were isolated from culture supernatants by (dUC). Macrophages secreted a large amount of sEVs in the first 24 h, with size ranging from 40–150 nm, peaking at 105 nm, as evaluated by nanoparticle tracking analysis and scanning electron microscopy. The markers Alix, CD63 and CD81 were detected by immunoblotting in EV samples, and the co-localization of CD63 and CD81 after sucrose density gradient ultracentrifugation (S-DGUC) indicated the presence of sEVs from late endosomal origin. Confocal fluorescence revealed that the sEVs were internalized by primary macrophages after three hours of co-culture. The methodology here applied aims to contribute for enhancing reproducibility between the limited number of available protocols for the isolation and characterization of MDM-derived sEVs, thus providing basic knowledge in the area of EV methods that can be useful for those investigators working with sEVs released by human primary macrophages derived from circulating monocytes.

## Introduction

Cells can communicate with each other through the secretion of extracellular vesicles (EVs) [1], which are small membrane-limited particles composed by a phospholipidic bilayer naturally released in the extracellular environment by different types of cells [2, 3]. These vesicles are present in many biological fluids, including amniotic and cerebrospinal fluids, blood, breast milk, urine, saliva, and semen [4–10]. Accumulated evidence has demonstrated that EVs participate in cell-to-cell communication through the transport of bioactive molecules, such as antigen-presenting molecules, membrane receptors, proteins, lipids, cytokines, DNA, RNA, mRNAs, and microRNAs [11–13]. The EV-mediated intercellular signaling contributes to the regulation of pathological or physiological cell processes, such as angiogenesis, maintenance of homeostasis, cardiovascular diseases, cell signaling, inflammation, and cancer [13–15]. Moreover, EVs are taken up by macrophages, thus functioning as a vehicle to exchange components among cells of the immune system and to strengthen the immune response against pathogens [16–18].

Although in recent years there has been much progress on the understanding of the fundamental biology of EVs, some aspects related to their biogenesis, secretion mechanisms, interaction with recipient cell and function are not yet clear [13, 19]. One reason is that current protocols for EV isolation do not guarantee the purification of specific EV subtypes, resulting in a mixture of heterogeneous vesicles derived from different subcellular fractions. This limitation hampers a better understanding of the role of a given EV subtype in specific physiological or pathological processes [19, 20]. Therefore, it is essential to know the physical and biochemical characteristics of EVs through several analytical methods that allow assessing their purity, integrity, concentration, and interaction with recipients cells before evaluating the functional properties of these vesicles [13, 21].

Currently, EV types may be classified according to their intracellular origins (endosomal system or plasma membrane), sizes, and density ranges [19]. Based on their size, EVs are subdivided into three groups: large EVs (LEVs, >300 nm in diameter), derived from the outward budding and fission from the plasma membrane of apoptotic or healthy cells [22, 23]; intermediate size EVs (IEVs, 150–300 nm), and small EVs (sEVs, <150 nm), originated from endosomal or non-endosomal systems [19, 20]. sEVs from the endosomal system have been shown to be originated from intraluminal vesicles (ILVs) contained in cytosolic multivesicular bodies (MVBs), which later fuse with the plasma membrane releasing them in the extracellular environment [24, 25]. Moreover, EVs can be co-isolated with other particles, like exomeres and lipoproteins from various densities (high, intermediate and low) when using differential ultracentrifugation (dUC) method [19, 26]. Given that apoptotic bodies are released from cells during the process of apoptosis [27, 28], here the term extracellular vesicles (EVs) will be used to refer only to vesicle preparations containing intermediate and small EVs obtained from healthy cells.

Most of the studies about EVs from human cells have been made with cell lineages, including HeLa [29, 30], THP-1 [18, 31], HEK [32, 33], HMC-1 [34, 35], intestinal cell lines [36], or brain endothelial cells lines [37, 38], and few with primary cells, such as dendritic cells (DCs) [39–41] or neutrophils [42]. However, detailed protocol for isolation, characterization, and analysis of interaction with recipient cells of sEVs released by primary human macrophages derived from circulating monocytes are barely available.

Macrophages play essential roles in the activation and modulation of innate and adaptive immune responses against a repertoire of pathogens, including bacteria, protozoan parasites, fungi and viruses, thus critically contributing for the preservation of the host physiologic homeostasis throughout life [43, 44]. Moreover, macrophages participate in many physiologic processes, such as brain development [45], bone remodeling [46], erythropoiesis [47], tissue regeneration [48], and the interaction between the immune and neuroendocrine systems [49]. Because few works have described the characterization of EVs from human primary macrophages, and taking into account that the adherence to rigorous criteria for EV isolation is essential for obtaining reliable experimental results, we present here an alternative protocol, based on classical dUC method, for isolation and characterization of small EVs released by human primary macrophages derived from circulating monocytes, and for their interaction with recipient macrophages.

## Materials and methods

### Ethics statements

The experimental procedures involving human cells in this study were performed with samples obtained after written informed consent and were carried out under the guidelines and regulations approved by the Research Ethics Committee of the Oswaldo Cruz Institute/Fiocruz (Rio de Janeiro, RJ, Brazil) under the number 397–07.

### Culture of human primary macrophages

Human monocyte-derived macrophages were obtained from buffy coats of healthy human blood donors, as previously described [50]. In summary, peripheral blood mononuclear cells (PBMCs) that had been isolated by density gradient centrifugation (Ficoll-Paque Premium 1.077; GE Healthcare Biosciences) were plated (4.0 x 10^7^ cells in 4 mL of medium) onto three 25 cm^2^ flasks (Greiner Bio-One) in Dulbecco’s modified Eagle’s medium (DMEM; LGC Bio) containing 10% human serum (Merck Millipore) and penicillin-streptomycin (LGC Bio). Cells were maintained at 37 °C in 5% CO_2_ for 7–10 days for monocyte differentiation into macrophages. Non-adherent cells were washed out with sterile phosphate-buffered saline (PBS), and the remaining macrophage layer was maintained in DMEM with 5% human serum. Macrophage purity was >90%, as determined by flow cytometry (FACScan; Becton Dickinson) analysis using anti-CD3 (BD Biosciences) and anti-CD68 (Southern Biotech) monoclonal antibodies.

### Isolation of sEVs

The macrophage layer was extensively washed with PBS and thus replenished with 10 mL of fresh medium supplemented with 5% EVs-depleted fetal bovine serum (FBS; ThermoFisher; from now on referred to as EV-free medium) and cultured for 24h, 48h or 72h. Then, culture supernatants were collected, transferred to 15 mL conical sterile polypropylene centrifuge tubes, and underwent progressive centrifugation steps to isolate the sEVs, as previously described [51], with minor modifications (S1 Fig). In brief, isolation was set up as follows: 400×g for 10 min to remove floating cells; 2,000×g for 10 min to eliminate dead cells and cell debris; 18,000×g for 40 min to remove some LEVs (including apoptotic bodies and other vesicles >300nm); then, the supernatants were transferred to 13 mL polypropylene tubes and submitted to 130,000×g for 70 min to pellet EVs; finally, the pellet-containing IEVs and sEVs was washed once with PBS at 130,000×g for 70 min. The final pellet was resuspended in PBS (50 μL) and maintained at -80 °C for upcoming assays. High centrifugation steps (18,000–130,000×g) were carried out using an SW41 Ti titanium swinging-bucket rotor in an Optima XE-90 centrifuge (Beckman Coulter). Next, isolated sEVs were characterized by nanoparticle tracking analysis (NTA), scanning electron microscopy (SEM), western blot and confocal microscopy, as described below.

### Cell viability assay (XTT-based cytotoxicity assay)

Macrophage viability was measured using tetrazolium salts (XTT), Cell Proliferation Kit II (Sigma-Aldrich) according to the manufacturer’s instructions. Cells were then cultured with EV-free medium for 24, 48 or 72h, after which the XTT assay was performed. Cell proliferation was spectrophotometrically quantified using a 96 wells plate reader at 450 nm. A decrease in optical density was analyzed by normalization against untreated cells with EV-free medium (control cells). All assays were prepared in triplicates.

### Protein extraction and quantification assays

Cellular proteins were extracted by homogenization with 200 μL RIPA Lysis buffer (Sigma-Aldrich) containing protease inhibitor cocktail 1:100 (cOmplete^™^, Sigma-Aldrich). For sEV proteins, samples (50 μL) were lysed by adding RIPA buffer (30 μL) with a protease inhibitor cocktail and then incubated on ice for 10 min. Samples were sonicated (frequency 60 Hz) in water bath three times for 5 min and vortexed (1 min) between each cycle to ensure protein homogenization and membrane lysis. For protein quantification, DC Protein assay (Bio-Rad) was carried out according to the manufacturer’s instructions. Absorbance and readings were obtained at 750 nm on a microplate reader SpectraMax M2 (Molecular Devices), and data were analyzed by SoftMax Pro 6.1 software (Molecular Devices). In parallel, Qubit Protein assay (ThermoFisher) was performed using the Qubit 2.0. Fluorometer. Results obtained using both methods were compared.

### ZetaView nanoparticles tracking analysis

EVs sizes were measured using ZetaView nanoparticle tracking analyzer (NTA; Particle Metrix GmbH). For measurements, samples were diluted to 100, 500, 1000, and 2000 in previously filtrated PBS (0.22μm) for optimal concentration range for the NTA software (ZetaView Software version 8.02.31, Particle Metrix GmbH). Software parameters were the temperature at 23°C, the sensitivity of 30–85 frames per second (fps), a shutter speed of 55, and laser pulse duration equal to that of shutter duration. Acquisition parameters were set to a minimum brightness of 20, a maximum size of 200 pixels, and a minimum size of 5 pixels. Polystyrene particles (Microtrac GmbH) with an average size of 100 nm were used to calibrate the instrument before sample readings. Data were analyzed using ZetaView software and Microsoft Excel 2013 (Microsoft Corp).

### Scanning electron microscopy (SEM) analysis

Samples were prepared as previously described [52], with minor modifications. After the last ultracentrifugation, pellets containing sEVs were resuspended (50 μL) in 2.5% glutaraldehyde in cacodylate buffer (0.1 M), pH 7.2, and samples (10 μL) were adhered in glass coverslips, previously covered with Poly-L-lysine (Sigma-Aldrich). After 30 min at 37°C, coverslips were washed three times in cacodylate buffer and post-fixed with a solution of 1% OsO_4_, containing 0.8% potassium ferrocyanide and 5 mM CaCl_2_ for 20 min at 25°C. After new washings with the same buffer, samples were dehydrated in ethanol ascending series (50, 70, 90, 100 and 100%), dried using the critical point method, mounted on aluminum stubs, and finally coated with a 20-nm-thick gold layer, and examined with a scanning electron microscope (Zeiss Auriga 45–38, Zeiss).

### Western blotting

For assessing the protein profile of macrophages or sEV preparations, western blotting was carried out as described [53], with modifications. After protein extraction, samples were resuspended in lithium dodecyl sulfate (LSD) buffer (Life Technologies) with or without reducing agent (Life Technologies), when applicable. Samples (40 μg of protein) were boiled at 70 °C for 10 min, loaded into 4–12% sodium dodecyl sulfate-polyacrylamide gel electrophoresis (SDS-PAGE) precast gels, and transferred to polyvinylidene fluoride (PVDF, ThermoFisher) membranes, which were blocked with 5% skimmed milk in Tris-buffered saline-Tween 20 (TBS-T, 0.01%) for 1h at room temperature. Blots were incubated for 18 h at 4 °C with anti-Alix, anti-CD63, anti-CD81, anti-Calnexin, anti-Cytochrome C, and anti-β-actin (control) antibodies. After washing with TBS-T, the membrane was exposed to secondary antibodies conjugated to horseradish peroxidase (HRP) anti-mouse or anti-rabbit, as required, for 1h at room temperature, and washed again with TBS-T. Protein bands were revealed using Western Chemiluminescent ECL Luminol substrate (GE Healthcare), and images were captured by C-DiGit Blot Scanner (LI-COR Biosciences). Relative band intensity was calculated using ImageJ software (NIH, USA). The antibodies used for western blotting assay, including their dilutions and supplier, are described in Table 1. Of note, the sEV protein markers were selected taking into account the minimal requirements recommended by the International Society for Extracellular Vesicles [54].

**Table 1:** Primary and secondary antibodies used for Western blotting to identify sEVs markers.

Antibodies	Molecular weight	Origin	Dilution	Supplier	Catalog Number
Anti-Alix	95 kDa	Mouse	1:1000	Cell Signaling	2171
Anti-CD63	30–60 kDa	Mouse	1:1000	Thermo Fisher	10628D
Anti-CD81	25 kDa	Mouse	1:500	Thermo Fisher	10630D
Anti-Calnexin	90 kDa	Rabbit	1:1000	Santa Cruz	11397
Anti-Cytochrome c	15 kDa	Mouse	1:500	Santa Cruz	13156
Anti-β-Actin	42 kDa	Mouse	1:45.000	Sigma-Aldrich	A3854
Anti-mouse-HRP		Goat	1:2000	Cell Signaling	7076
Anti-rabbit-HRP		Goat	1:2000	Cell Signaling	7074

Supplier information, catalog number are described.

### Sucrose density gradient centrifugation

Purification of sEVs was performed using sucrose density gradient ultracentrifugation as previously described [55, 56], with modifications, whose detailed protocol is described in Supporting information.

### Interaction of sEVs with human primary macrophages

The protocols used to label sEVs and to evaluate their internalization by macrophages are described in Supporting information. Images of vesicle uptake by macrophages were taken at 63X under a laser scanning confocal microscope (Leica TCS SP8, Mikrosysteme GmbH).

### Data availability

We have submitted all relevant data of our experiments to the EV-TRACK knowledgebase (EV-TRACK ID: EV200058) [57].

### Statistical analysis

All statistical data were performed and analyzed using GraphPad Prism Software version 7.0. NTA, sEV protein kinetics and cell viability data were subjected to two-way analysis of variance (ANOVA) with Tukey *post hoc* correction for determining significant differences between conditions. Mann–Whitney test for comparison of both protein assays and sEV size was applied. Data are shown as the median and quartiles (1st and 3rd), and the differences between values were considered statistically significant when the *P*-value was ≤ 0.05.

## Results

### Isolation, size, and number of EVs

**Fig 1:**
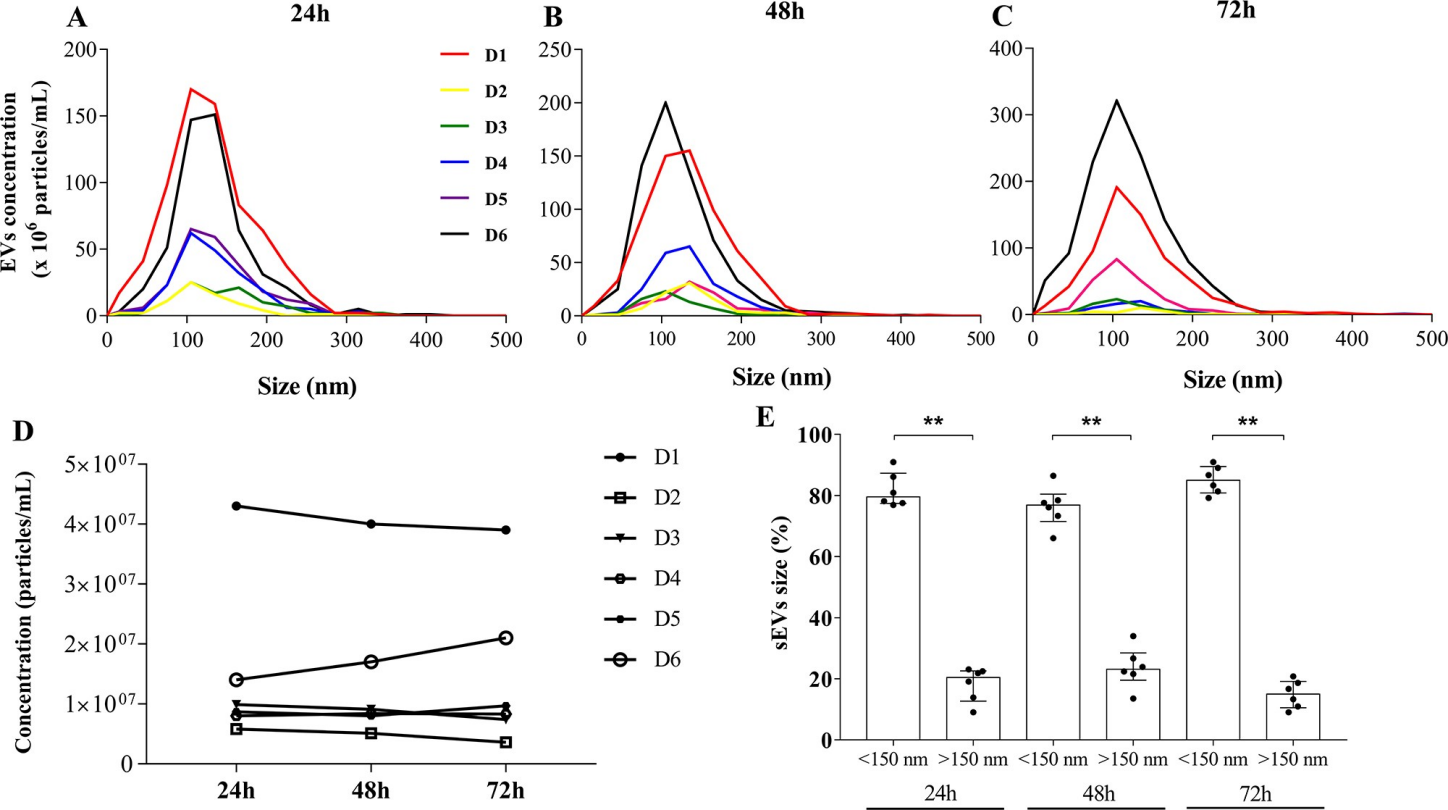
Concentration and size distribution of MDM-derived sEVs. NTA-ZetaView analyzes of total number and size distributions of EVs derived from six donors, isolated after (A) 24h, (B) 48h or (C) 72 h of cell culture. (D) Total concentration of EVs released at the same time-points (one culture flask for each point). (E) Proportion of EVs sizes <150nm or >150nm. Median values are indicated (*n* = 6). Mann–Whitney test was used to evaluate statistical significance: **p <0.01. D: donor.

Macrophages (ranging from 5.0 × 10^5^–1.3 × 10^6^/per flask) were cultured in EV-free medium during 24, 48, or 72h, and the EVs released after each of these time-points were isolated from culture supernatants by ultracentrifugation, as shown in (S1 Fig). The total number and size distribution of the isolated EVs were quantified in samples from six individual donors through NTA. According to NTA measurements, high concentrations of vesicles with size ranging between 40–150 nm, peaking at 105 nm, were obtained from cells cultured in the three different time-periods (Fig 1A–1C), despite a slight variation in the total number of vesicles among donors. It can also be observed that, except for D6, the concentration of vesicles harvested was nearly the same at the three time-points (Fig 1D), meaning that 24h is time enough for optimal shedding of EV-macrophages. The proportions of EVs with size <150 nm were equal to the median 79,54% (1st quartile: 77,39 and 3rd quartile: 87,31%), 76,86% (71,5 and 80,43), 85% (80,82 and 89,5) at the same time-periods, respectively (Fig 1E). Similar results were found with EVs obtained from macrophages of extra six donors (S2 Fig). The vesicle median size was equal to 110 nm, with a mode size of 105 nm (Table 2). Moreover, the XTT assay indicated that macrophage survival cultured in EV-free medium during 24, 48, or 72h was not affected. The proportions of viability were equal to the median 95,55% (93,55 and 100,8), 93,1% (89,71 and 100,5), and 91,03% (86,17 and 96,19) at the indicated time-periods, respectively (S2D Fig). Our data show that the proposed dUC protocol allows isolation of a high number of heterogeneous populations of MDM-derived EVs, and that the vast majority of vesicles isolated from individual samples fall within the size expected for sEVs (<150 nm).

**Table 2:** Sizes and concentrations of sEVs obtained from human primary macrophages.

Donor	Time	Total approximate cell number in three flasks ^a)^	Original concentration Particles/cm^3^ ^b)^	Concentration Particles/mL ^b)^	Median size (nm) ^b)^	Mode size (nm)
**D1**	24h	2,2 x 10^6^	4.3 x 10^9^	4.3 x 10^7^	116,2	105
	48h	1,8 x 10^6^	4.0 x 10^9^	4.0 x 10^7^	123,4	135
	72h	2,4 x 10^6^	3.9 x 10^9^	3.9 x 10^7^	121,3	105
**D2**	24h	2,0 x 10^6^	2.9 x 10^9^	5.8 x 10^6^	111,2	105
	48h	2,1 x 10^6^	2.6 x 10^9^	5.1 x 10^6^	128,6	135
	72h	1,9 x 10^6^	1.1 x 10^9^	3.6 x 10^6^	132,9	135
**D3**	24h	2,1 x 10^6^	9.9 x 10^8^	9.9 x 10^6^	137,3	105
	48h	2,7 x 10^6^	9.1 x 10^8^	9.1 x 10^6^	107	105
	72h	2,5 x 10^6^	7.4 x 10^8^	7.4 x 10^6^	114,4	105
**D4**	24h	2,0 x 10^6^	4.0 x 10^9^	8.0 x 10^6^	123,1	105
	48h	1,9 x 10^6^	4.2 x 10^9^	8.4 x 10^6^	122,8	135
	72h	2,3 x 10^6^	1.7 x 10^9^	8.3 x 10^6^	119,2	135
**D5**	24h	1,7 x 10^6^	4.3 x 10^9^	8.7 x 10^6^	123,3	105
	48h	2,3 x 10^6^	4.0 x 10^9^	8.0 x 10^6^	128,6	135
	72h	2,0 x 10^6^	1.9 x 10^9^	9.7 x 10^6^	104,1	135
**D6**	24h	2,4 x 10^6^	1.0 x 10^10^	2.1 x 10^7^	117,6	135
	48h	2,8 x 10^6^	7.8 x 10^9^	2.6 x 10^7^	113	105
	72h	3,1 x 10^6^	1.9 x 10^10^	6.3 x 10^7^	121,9	105

^a)^ Three flasks for each time-point

^b)^ Calculated by ZetaView Software version 8.02.31

### Protein quantification of MDM-derived sEVs samples

**Fig 2:**
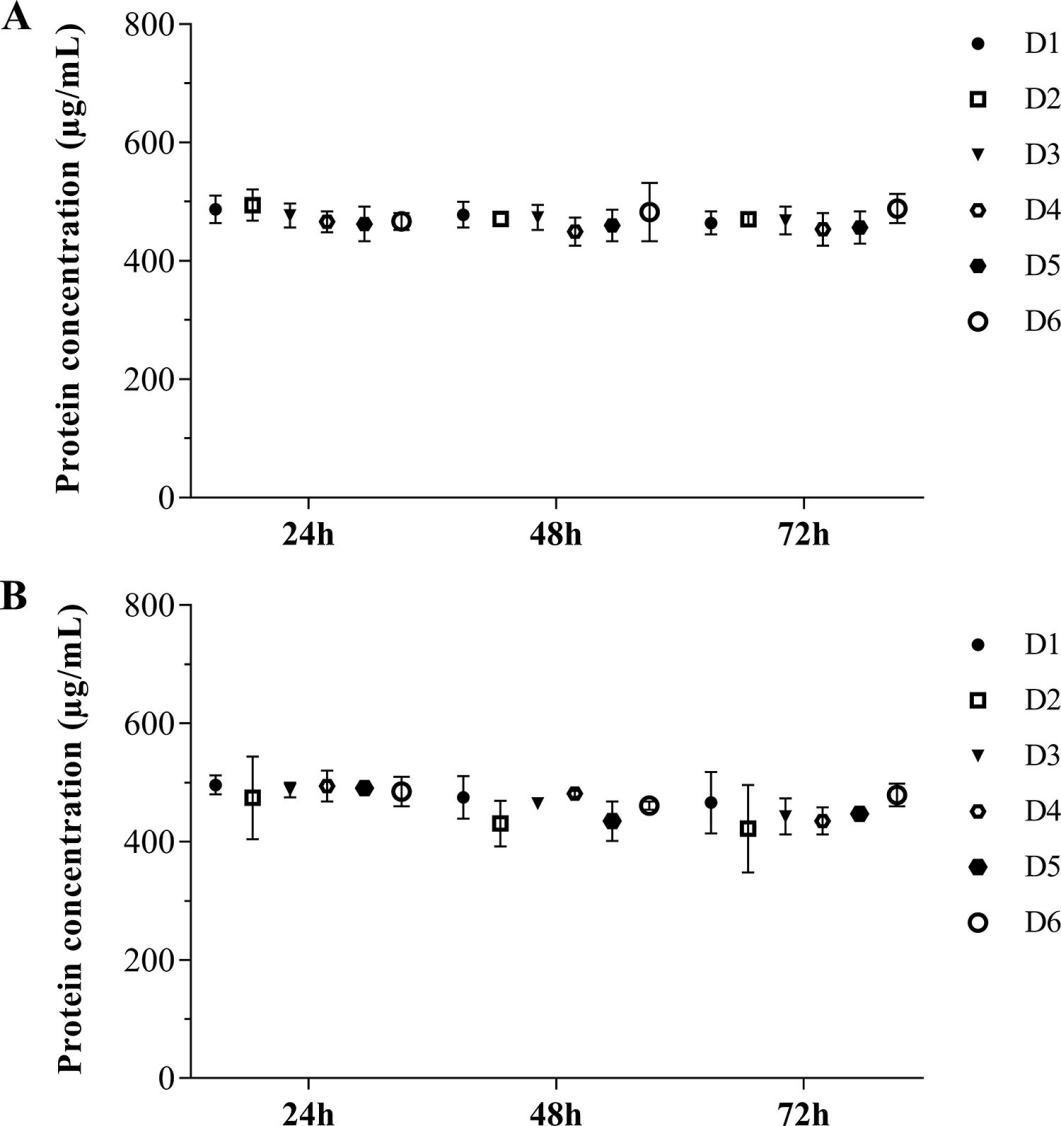
Protein concentration of MDM-derived sEVs. Protein concentration of sEVs obtained after 24, 48 or 72h of macrophage culture, measured by (A) Qubit or (B) DC protein assays (*n* = 6). Median values are indicated. D: donors.

Samples collected after 24, 48, or 72h of culture were analyzed by colorimetric (DC Protein) or fluorimetric (Qubit) assays to determine EV protein concentration with five μL of EVs as starting volume. All samples had ~500 μg/mL of total protein, with slight variations within the three time-points analyzed (Fig 2). To assure the accuracy of these results, we measured in parallel the protein amount of three known concentrations of bovine serum albumin (BSA), whereas no differences between both methods have been found (S3 Fig). We also measured the protein concentration in three other sEV samples, which provided similar results (S4 Fig). Thus, our analysis shows that both protein quantification assays provide reliable measurements of EV protein concentration. Of note, no significant variation was noticed in the protein content throughout the time points.

### Morphology and protein markers of MDM-derived sEVs

**Fig 3:**
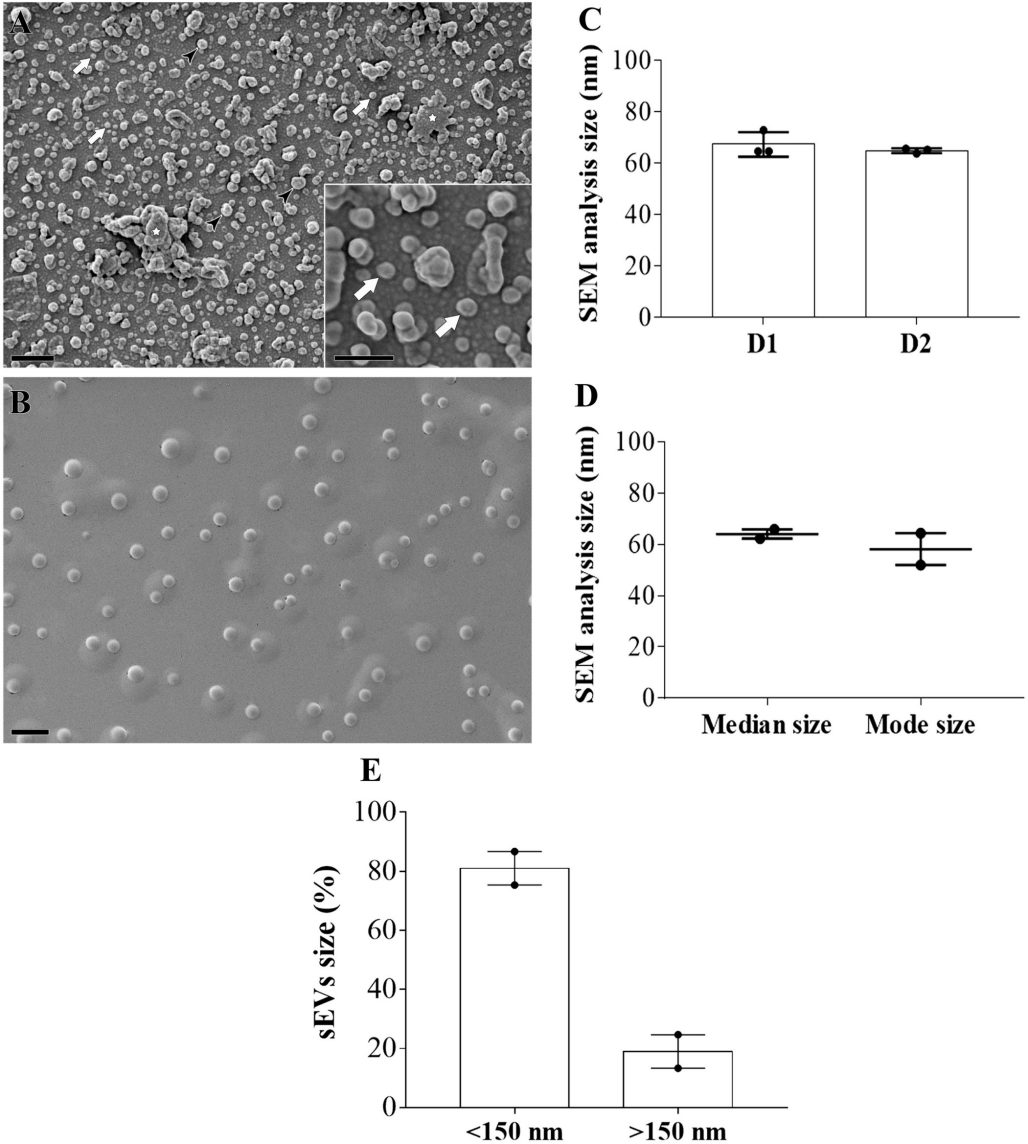
Morphological characterization of MDM-derived sEVs. (A) SEM microscopy of sEVs showing homogeneous vesicle-shaped structures with size ranging from 40–110 nm (Bars = 400 nm) (*n* = 2). White arrows point to sEVs with size around 100 nm, and black arrow heads point to vesicles with size >150 nm; white star shows clumps of sEVs. Inset shows 200 x magnification of sEVs (Bars = 200 nm). (B) Control image containing only fixation solution (Bars = 1000 nm). (C) Size measurement of sEVs from two individual donors (number of images analyzed per donor = 3). (D) Median and mode size of sEVs from the same two donors. (E) Proportion of EVs sizes <150nm or >150nm. Median values are indicated. D: donors.

**Fig 4:**
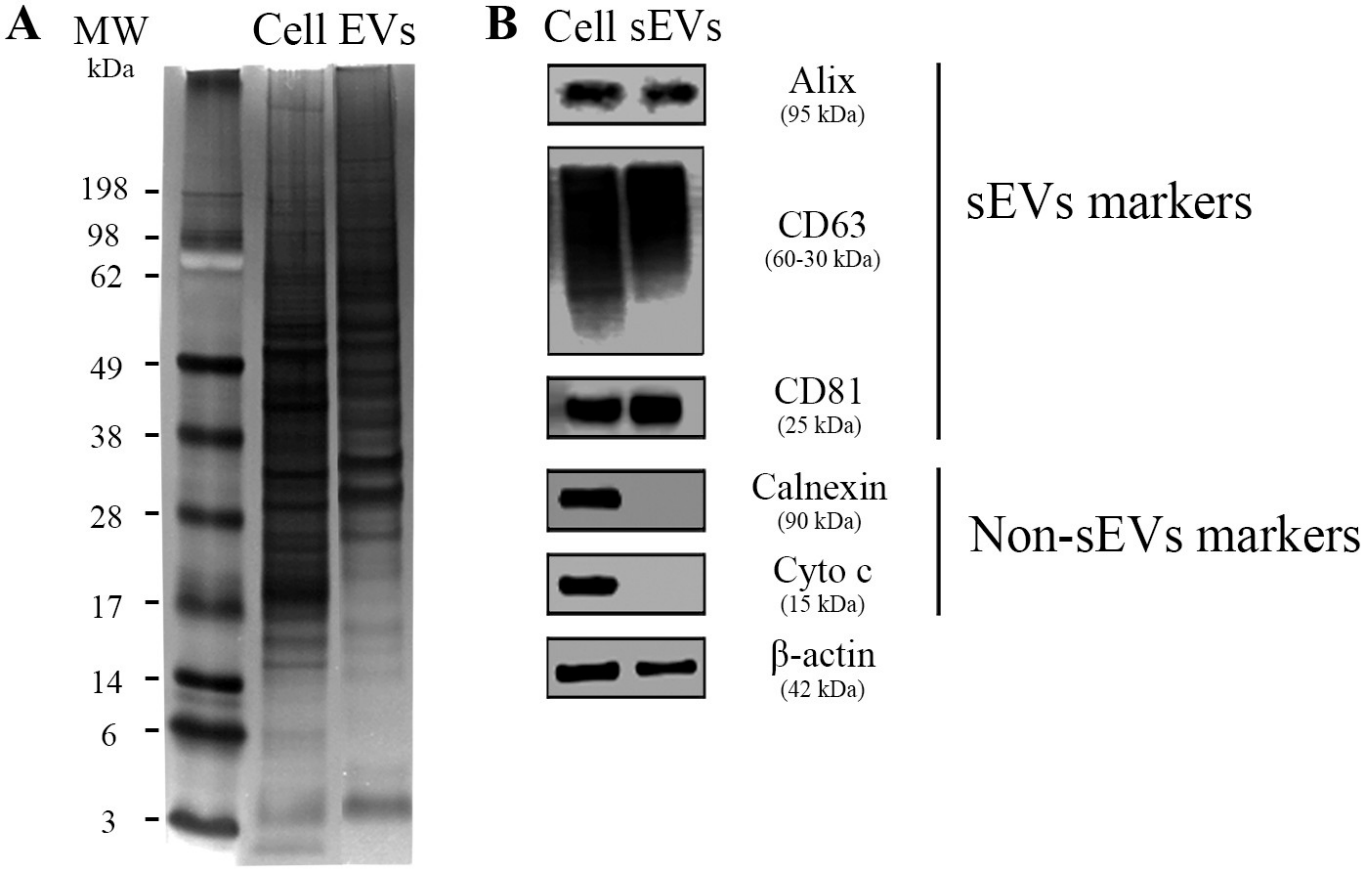
Protein markers of MDM-derived sEVs. (A) Representative image of three independent assays of polyacrylamide gel stained with silver nitrate after separation of 40 μg total protein from cell (Cell) or sEVs pool lysates (EVs; pools comprise samples from four individual donors). (B) Western blot analysis of sEVs markers (Alix, CD63, and CD81) and non-EVs markers (Calnexin and Cytochrome C) (*n* = 3). β-actin = loading control. 40 μg of total protein were loaded onto the gel. MW: molecular weight marker.

We next examined the nanoparticle morphology by SEM, which revealed the presence of sEVs with size ranging from 40–100 nm, as shown in Fig 3. Size measurements of sEVs from two individual donors (number of images analyzed per donor = 3) showed a median size of 64,6 nm (64,59 and 72,84 nm) (D1), 65,24 nm (63,8 and 65,59) (D2) and mode size of 64,34 nm and 51,98 nm, respectively (Fig 3C and 3D). The proportion of EVs with size <150 nm was 81% (75,33 and 86,67), and with size >150 nm was 19% (13,33 and 24,67) (Fig 3E). Aggregates or clumps of sEVs were observed in SEM analysis, as a result of vesicle isolation with high-speed centrifugation (Fig 3A) [58]. Next, the protein markers of these particles were identified by western blotting assays. We initially observed, as expected, that sEVs exhibited lower protein content compared to their parent cells, as macrophage lysates contained a strong protein expression initiating at 14 kDa, whereas the sEV lysates presented protein content from ~28 kDa (Fig 4A). Next, accessory proteins from the endosomal sorting complex required for transport (ESCRT) and tetraspanins proteins, such as Alix (95 kDa), CD63 (a 30–60 kDa glycosylated protein), and CD81 (25 kDa), were detected in the vesicles as well as in the whole cell lysates (control) (Fig 4B). The absence of markers for cytochrome c (mitochondria) and calnexin (endoplasmic reticulum) in the vesicle lysates indicates that EV preparations were not contaminated with components of the mitochondria and endoplasmic reticulum from dead cells. The complete blotting membranes are shown in S5 Fig. Thus, we identified vesicles with homogeneous round shape morphology in our samples, which are enriched with proteins from multi-vesicular endosomes (MVE), features that suggest the presence of sEVs from endosomal origin in our preparations.

### Separation of sEVs by sucrose density gradient

**Fig 5:**
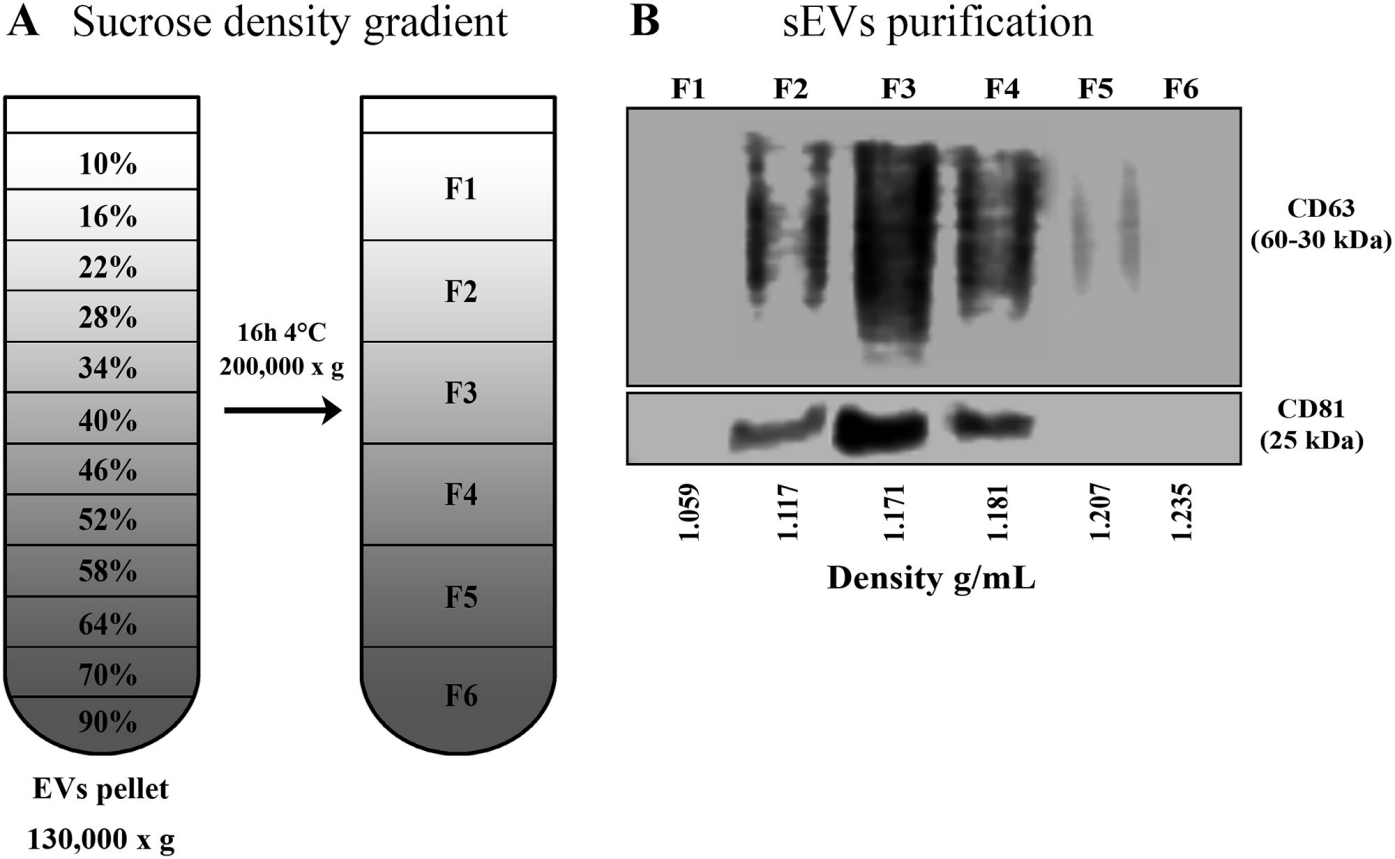
Sucrose density gradient ultracentrifugation in MDM-derived sEVs. (A) Separation of sEVs by sucrose density gradient. The final sEVs pellet 130,000 x g was placed onto 90–10% sucrose gradient layers, then centrifuged for 200,000 x g for 16h, as indicated. Six fractions were collected from the top to the bottom of the gradient for further WB and confocal experiments. (B) Western blot analysis of sEVs recovered at the fractions F1-F6 (*n* = 3). 15 μg of total proteins were loaded onto the gel. Relative band intensity was calculated by ImageJ.

To confirm the presence of sEVs from endosomal origin, sucrose density gradient ultracentrifugation (S-DGUC) assays were performed (Fig 5A and S6 Fig). The original pellets from the 130,000 x g spin were then fractionated by S-DGUC at 200,000 x g (Fig 5A), and the resultant fractions, numbered F1 to F6 (which were formed by the contiguous sucrose layers, as detailed in supplementary information) from the top to the bottom of the tube, were subjected to blotting analysis for the EV markers CD63 and CD81. The blotting results were analyzed upon normalization of band intensities, as described elsewhere [61]. We found that CD63 and CD81 were predominantly present in the middle-density gradient F3 (49,28% and 54,92% of the total signal, respectively). Moderate intensity amount of CD63 was detected in F4 (29,82%) and F2 (18,54%), whereas signal reduction was noticed in F5 (2,35%). CD81 labelling F2 (20,997%) and F4 (23,05%) was less intense than F3 (Fig 5B). The raw blotting images from gradient assays are shown in S6 Fig. Our data showed that CD63 colocalized with CD81 in fractions F2 to F4 (densities between 1,117 to 1,181 g/mL), a buoyant property reported for sEVs from endosomal origin [59, 60].

### Internalization of sEVs by human primary monocyte-derived macrophages

**Fig 6:**
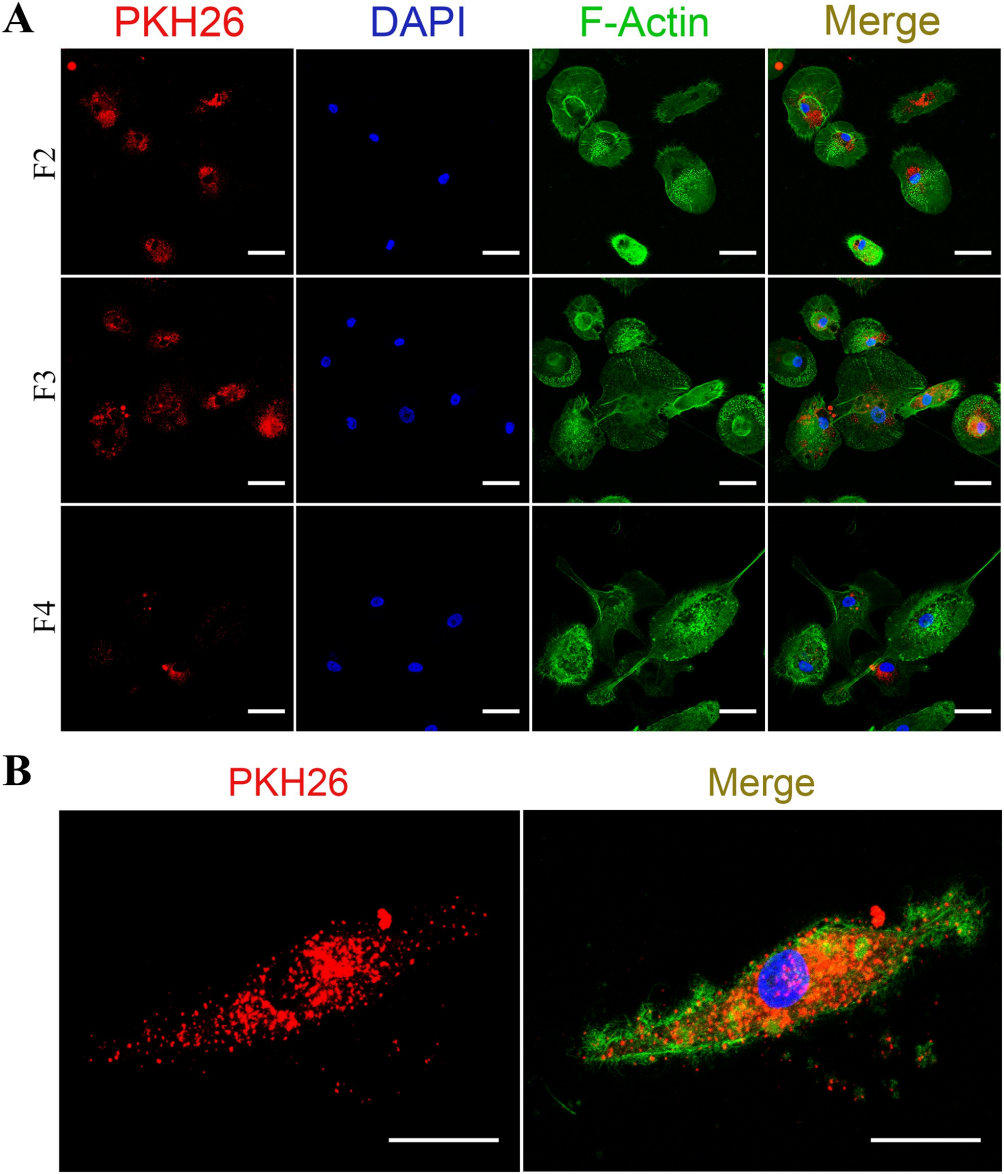
Internalization of sEVs by recipient human primary macrophages. (A) sEVs labeled with PKH26 were separated by sucrose density gradient ultracentrifugation and the fractions F2-F4 were added to recipient macrophages during 3 hours (*n* = 3) (Bars = 50 μm). (B) representative image of a single macrophage (5x magnification) that internalized labeled sEVs from F3 (Bars = 25 μm). PKH26: sEVs; DAPI: cell nuclei; F-Actin: macrophages.

**Fig 7:**
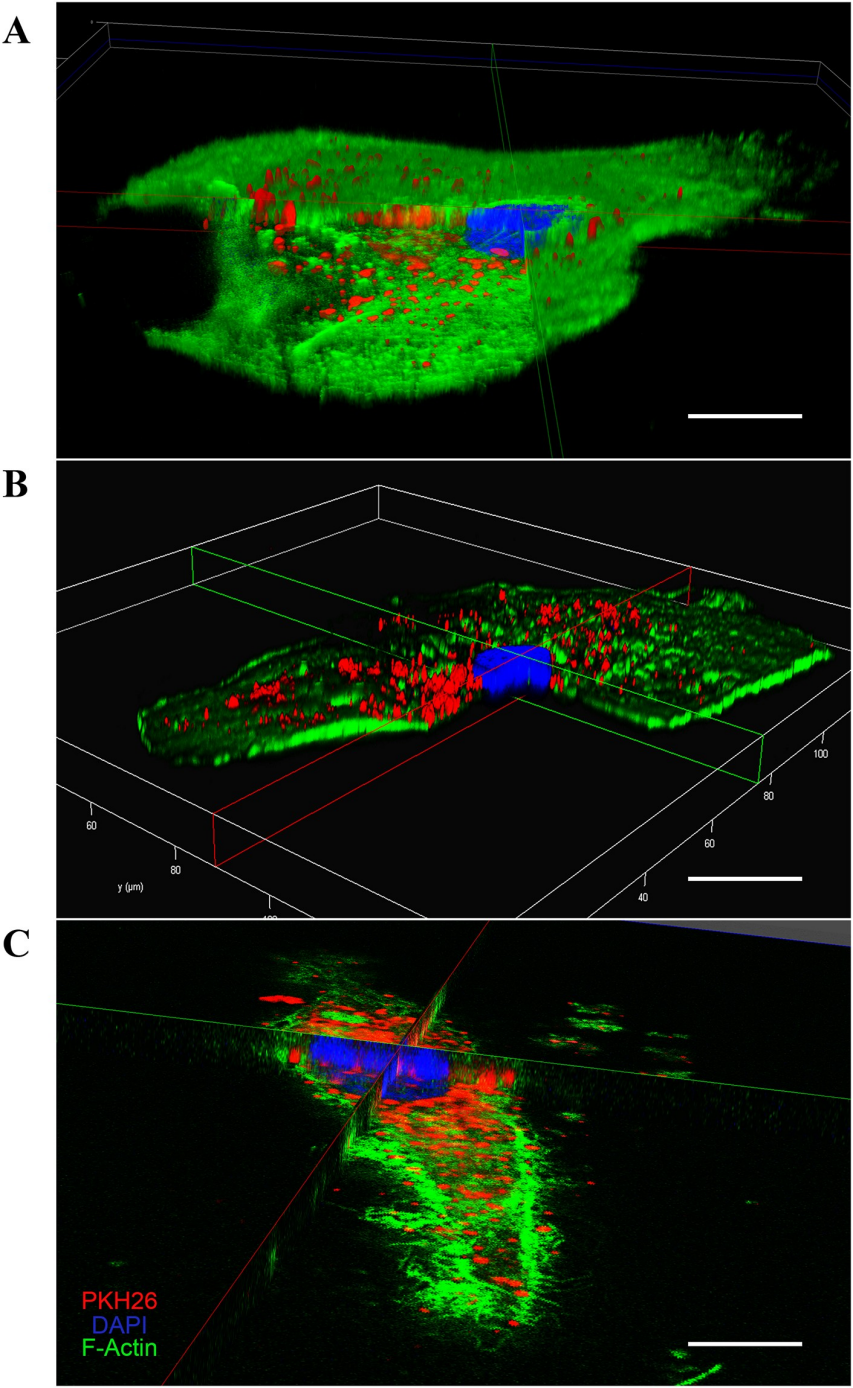
Internalization of sEVs by recipient human primary macrophages. 3D reconstruction image (10X magnification) showing uptake by macrophage of non-purified sEVs after (A) 15 min, (B) 30 min or (C) of sucrose gradient-purified sEVs from fraction F3 after 3h of interaction (*n* = 3). Scale bar = 25 μm.

Next, we analyzed, by confocal microscopy, whether the sEVs would be internalized by macrophages. To this end, sEVs were firstly labeled with the lipophilic dye PKH26 (PKH26-labeled EVs, as described in Supplementary methods) and then fractioned by sucrose gradient. Next, the PKH26-labeled sEVs from fractions F2, F3 and F4 were added separately to recipient macrophages, and the preparations were incubated for 3h. Images show that PKH26-positive vesicles were internalized by macrophages, as evidenced by the red puncta observed in the cells stained with green phalloidin and DAPI (Fig 6). Images of control macrophages treated with EV-free medium labeled with PKH26 (S7 Fig) show a diffuse fluorescence in F4 (PKH26 and merge columns), probably representing contaminants (e.g., lipoproteins, protein complexes, ribonucleoproteins) and/or PKH26 micelles. We also analyzed the interaction of sEVs present in the original pellets without gradient separation, for different periods of times. We observed that macrophages were able to internalize particles as prompt as no more than 15 or 30 min of interaction (S8 Fig), while, as expected, macrophages engulfed a more substantial number of particles after three hours of co-culture. Importantly, the internalization of purified or non-purified PKH26-labeled sEVs by macrophages were confirmed by 3D reconstruction (Fig 7). Thus, our data show that primary macrophages readily interact with and internalize sEVs emitted by other macrophages, and that the presence of some contaminants in the non-purified EV preparations did not impair the uptake of sEVs.

## Discussion

We report here a methodical approach for isolation, enrichment and characterization of sEVs released by human primary macrophages from circulating monocytes in culture supernatants. We performed this work taking into account the limited number of studies describing procedures for recovering sEVs from those cells. Due to growing evidence of the critical role of macrophages in a variety of physiological and pathological conditions [43, 44], along with the ability of EVs to mediate intercellular communication, the application of reliable methods for the separation of EVs may contribute for a better understanding of the biology of EV-based cellular communications in the macrophage functions. In fact, the use of standardized methods to provide pure populations of EV subtypes is critically important to define the biological functions of EVs in multicellular organisms, as pointed out by other authors [54, 61, 62]. Therefore, several groups in the field are exploring possibilities to improve or design new methods for EV isolation and characterization that allow the understanding of their biogenesis, targeting and physiological role [63].

To date, there are no published protocols detailing step-by-step the isolation and characterization of small EVs secreted by human macrophages derived from circulating monocytes. Available studies about MDM-derived sEVs (Table 3) have applied different procedures for their description, were mainly aimed to functional approaches and not to methodological improvements, or evaluated multiple proteins or microRNA contents from a heterogeneous population of vesicles secreted by macrophages. In fact, some of these works used large volume of culture supernatant or even additional isolation steps before the final centrifugation (e.g., filtration) [17, 18, 64–72]. In contrast, we suggest here a modified protocol based on sucrose density gradient ultracentrifugation with higher speeds that allowed by itself the separation and quantification of large amounts of small EVs enriched with vesicles from the endosomal origin, which is consistent with results reported by other authors [51], who showed that dUC with increasing speed pelleted EVs with decreasing sizes. Furthermore, many studies related to sEVs from human macrophages used the monocytic leukemia cell line THP-1 [18, 31, 73, 74]. The different features between primary and tumor cell lines do not allow a fair comparison of the methods used for isolation and characterization of sEVs from primary cells and their corresponding tumor lines, thus highlighting the importance of applying specific protocols for primary macrophages.

**Table 3:** Available studies* of EVs secreted by primary human macrophages derived from circulating monocytes.

Research objective	EV isolation methods	EV characterization methods	Filtration as an extra isolation step	Internalization assay	References
Functional study	UC	TEM, FC, WB and Sucrose gradient assay	No	No	[65]
Functional study	UC	TEM, LP, Sucrose gradient assay and PT	Yes	Yes	[66]
Proteomic study	UC	NTA, TEM, WB and PT	Yes	No	[67]
Proteomic study	UC	TEM, WB and PT	Yes	No	[17]
Micro RNA profile	UC	Iodixanol gradient assay, AChE activity and WB	No	No	[68]
Functional study	EVs isolation kit	NTA, TEM and WB	No	No	[70]
Functional study	EVs isolation kit	TEM and WB	Yes	No	[69]
Functional study	UC	NTA, TEM, FC and WB	Yes	No	[18]
Functional study	UC	NTA, TEM and WB	Yes	No	[71]
Functional study	UC	NTA, TEM and WB	Yes	No	[64]
Functional study	UC	NTA, TEM and FC	No	No	[72]

AChE: acetylcholinesterase activity; LP: Lipidome; FC: Flow citometry; PT: Proteomic

*PubMed, June 2020

Here, MDM-derived sEVs from healthy human donors were characterized according to their physical and biochemical properties, as well as their interaction and internalization by recipient macrophages. Based on S-DGUC, other authors have considered that sEVs include particles with sizes between ~50–150 nm [19, 20, 59]. According to NTA measurement, our sEVs were in the same size range, peaking at 105 nm. In the method here described, the vesicle median sizes for all donors analyzed were mostly uniform, fluctuating within the sEVs dimensions already described [59], although slight variations in the total number of vesicles from individual samples were observed. Unlike the donor-to-donor variability in particle concentrations, the number of sEVs from individual donors did not fluctuate throughout the days the sEVs were collected. All samples produced very similar NTA profiles, indicating data reproducibility at these conditions.

Previous findings suggest that EV yield and protein concentration may depend on several factors, including cell type, cell confluence level, cell activation by exogenous compounds (e.g., Ca^+2^ ionophores, cell detachments, hypoxia, etc) [75–77] and culture conditions [40, 78, 79]. In this sense, some specifics of our culture conditions, such as small variations in the number of macrophages (see Table 1) and time of culture (24, 48 or 72 h) probably did not influence the total protein concentration of sEVs, as ascertained by the use of two different protein quantification methods. Moreover, in addition to the conventional assays, such as DC Protein, which is based on the Lowry method, we also confirmed that the Qubit assay is a reproducible and reliable method for measuring proteins of EV samples, which is consistent with previous results of Vergauwen et al, [80], who measured protein concentration of EVs derived from the epithelial breast cancer cell line MCF-7.

The scanning electron microscopy (SEM) analysis revealed a homogeneous population of small EVs with round shape morphology and size smaller than 100 nm, features also found reported in other EV studies [81, 82]. The small amounts of LEV (vesicles larger than 150 nm) detected in our preparations were also expected, since it has been described that separation of EVs by high speed dUC resulted in a heterogeneous population of EVs with different sizes and subcellular origins [20]. Furthermore, NTA and SEM measurements detected a higher proportion (~81%) of vesicles with size <150nm than with size >150nm (~19%), thus suggesting a high enrichment of MDM-derived sEVs with the present protocol.

The identity of vesicles in our samples was further defined by evaluating the expression of small EVs protein markers, such as Alix, CD63, and CD81. These molecules were detected in all samples. Additionally, samples did not express non-EV markers, such as calnexin and cytochrome C, showing that vesicle preparations were not contaminated with components of the mitochondria or endoplasmic reticulum derived from cellular debris. The blotting assays were performed according to recommendations of the International Society for Extracellular Vesicles (ISEV) for an appropriate use and precise documentation for methods related to EV research [54], such that we used three categories of proteins present or enriched in EVs (one cytosolic and two membrane-bound proteins) and two other global proteins «not expected in EVs» (such as mitochondria and Golgi proteins).

To achieve a better specificity of EVs or EV subtype separation, we applied an additional purification technique based on density gradients. We selected the sucrose density gradient taking into account its property to separate membrane-limited vesicles based on their floatation speed and equilibrium density [83, 84]. Moreover, this technique has been proved to be a robust approach for EV purification for consistent functional and structural analyses [20, 59]. Western blot analyses revealed markers for late endosome proteins (CD63 and CD81) in the fractions with low and middle-density gradients (F2-F4; 1.117 to 1.181 g/mL), which is consistent with a previous report that reasoned that the tetraspanins CD63, CD9, and CD81 identify sEVs of endosomal origin from primary dendritic cells [59]. Although we have not used the CD9 marker, we detected the presence of ESCRT-accessory molecules Alix and the colocalization of CD63 and CD81 after sucrose density gradient. Therefore, we propose that the separated preparations after S-DGUC contain sEVs from late endosomal system.

We also found, through confocal microscopy, that recipient macrophages uptake sEVs with three hours of co-culture, whereas other studies reported that internalization occurred after more extended periods (12-24h) [37, 66, 85]. Other authors may have opted for longer co-culture times to ensure the visualization of the internalized sEVs. In our work, the same origin (human macrophages) and nature (primary cells) of emitting and recipient cells may have contributed to the rapid uptake of the vesicles [13, 86]. Large fluorescent dots were observed in some fractions containing positive PKH26-labeled sEV or control PKH26-labeled sEV-free medium (an unspecific diffuse fluorescence in F4), suggesting the presence of some contaminants (e.g., aggregates, lipoproteins, protein complexes, ribonucleoproteins). This effect could be a consequence of high-speed centrifugation of culture medium in combination with lipophilic dyes such as PKH, which induce the formation of artifacts with different sizes and morphologies that can be detected by fluorescence microscopy [58, 87, 88]. The specialized literature has also reported a multitude of contaminants in vesicles separated by multi-step methods [60, 89, 90]. Moreover, we believe that staining EVs with diameter smaller (<200 nm) than the diffraction limit of light of the confocal microscopy may also have potentially favored the visualization of other large fluorescent dots in our preparations, probably corresponding to clusters of positive PKH26-labeled sEV without the possibility to discriminate one vesicle from another [91, 92]. To isolate highly purified EVs simultaneously with the depletion of non-EV material from a given biofluid or cell conditioned media is difficult using the available tools existing today. In other words, separating sEVs from contaminants that may share biophysical properties with EVs is still a major challenge in the field [60, 89].

During the processes of MDM-derived EV isolation using S-DGUC, there were some methodological advances that should be reported in the modified protocol. First, the protocol permitted us to obtain ~80% of heterogeneous population of sEVs (<150nm) using intermediate samples sizes (10 mL). Second, the proposed method showed reproducibility between macrophage samples from different healthy donors, obtaining sEVs with similar physical and biochemical characteristic according to the analyzes of NTA, SEM and WB. Finally, this is the first study to report the co-localization of CD63 and CD81 markers in the fractions subjected to imunoblotting, indicating the presence of late endosome-derived sEVs of human primary macrophages derived from circulating monocytes.

In conclusion, given the technical difficulties in the EV field, the use of appropriate methodologies for obtaining EVs is critical for understanding their biogenesis and role in cellular communication processes. The protocol that we applied, combining dUC with density gradient purification assays, allowed the isolation of small EVs of endosomal origin released by human primary macrophages. One limitation is necessary to be considered in this study, as we did not use markers to identify the contaminants present in our EVs isolated after the density gradient assay. Since several contaminants have been identified in EVs separated by multi-step methods [90], determining the presence of certain contaminants may also be necessary for specific functional applications of the MDM-derived sEVs. Finally, our work seeks to contribute for enhancing the reproducibility between the limited number of available protocols for the description of MDM-derived sEVs, thus providing an alternative methodology research groups working with EVs released by these cells.

## Supporting information

S1 File (DOCX)

S1 Fig Centrifugation steps for isolation of MDM-derived sEVs. The flowchart shows the centrifugation steps applied for sEVs isolation from 10 mL of supernatants from monocytes-derived macrophages cultured in DMEM with 5% EVs-depleted serum. MØ: Macrophage; LEVs: large extracellular vesicles; IEVs: intermediated extracellular vesicles; sEVs: small extracellular vesicles.
(TIF)

S2 Fig Concentration, size distribution of sEVs and macrophages viability. EVs were colleted at 72 h of macrophages culture. (A) NTA-ZetaView analyzes of total number and size distributions of EVs derived from other six donors not represented in the main figures (*n* = 6). (B) Total concentrations of EVs released by different donors. (C) Proportion of EVs sizes <150 nm or >150 nm. Mean values ± SD are indicated. **p <0.01 (Mann–Whitney test). (D) Macrophages viability at different time periods (*n* = 4). Decrease in survival was analyzed by normalization against untreated cells with EV-free medium (control cells), represented by red dotted line. Median values are indicated D: donor.
(TIF)

S3 Fig Bovine serum albumin (BSA) quantification. BSA concentrations at (A) 600 μg/mL, (B) 400 μg/mL, and (C) 200 μg/mL were assessed by Qubit or DC Protein assays (*n* = 3). Identical sample volume (5 μL) were used in all assays. Red dotted line indicates known BSA concentration. Results are expressed as median. D: donor.
(TIF)

S4 Fig Protein concentration of MDM-derived sEVs. sEVs samples (**A**) 1, (**B**) 2, and (**C**) 3 from other donors colleted at 72 h of cell culture were measured by Qubit or DC protein assay (*n* = 3). Results are expressed as median.
(TIF)

S5 Fig Protein markers of MDM-derived sEVs. Raw blots of sEVs markers using 40 μg of total protein and specific antibodies for sEVs (Alix, CD63 and CD81) and non-sEVs (Calnexin and Cytochrome C) (*n* = 3). β-actin was used as charge control. All experiments were performed with pools (4 donors) of sEVs.
(TIF)

S6 Fig Raw blots images of fractions F1-F6 collected of MDM-derived sEVs separated by S-DGUC (*n* = 3). Polyacrylamide gels were loaded with 15 μg of protein and membranes were labeled with sEVs markers (CD63 and CD81). All experiments were performed with pools (4 donors) of sEVs.
(TIF)

S7 Fig Interaction of EVs-free medium with recipient primary macrophages. EVs-free medium were labeled with PKH26, then separated by sucrose density gradient centrifugation and three fractions (F2-F4) were colleted and added separately to recipient macrophages during 3 hours (*n* = 3). Fluorescent images represent only cells or cells with sEVs, respectively (Bars = 50 μm). PKH26: sEVs; DAPI: cell nuclei; F-Actin: macrophages.
(TIF)

S8 Fig Internalization of original EVs pellets by macrophages. EVs pellets isolated by dUC (not purified by sucrose gradient) were labeled with PHK26 and added to macrophages during 15 min (*n* = 3), 30 min (*n* = 3), and 3 hours (*n* = 4). Bright field (DIC) and fluorescent images represent only cells or cells with extracellular particles, respectively (Bars = 50 μm). PKH26: EVs and other particles; DAPI: cell nuclei; F-Actin: macrophages.
(TIF)

S9 Fig Protocol to layer sucrose density gradient. (A) Position of tube angled 90° and (B) angled 60° during layering sucrose gradients. (C) Correct and (D) incorrect sucrose gradients layers. Black arrows point to layer formation during sucrose gradient; black arrowheads point to diffuse layers.
(TIF)

S1 Raw image (PDF)
