# Supplementary material for: Characterization and internalization of small extracellular vesicles released by human primary macrophages derived from circulating monocytes
Source: PLoS One. 2020 Aug 24;15(8):e0237795. doi: 10.1371/journal.pone.0237795 (PMC7444811; doi:10.1371/journal.pone.0237795)
Supplement: S1 File — (DOCX) [file pone.0237795.s001.docx]

**Supplementary materials and methods**

1. **Equipment**
2. **For sucrose density gradient ultracentrifugation (**S-DGUC**):**

- Ultracentrifuge (e.g., Beckman Optima XE-90 centrifuge)
- Ultracentrifuge rotor (e.g, SW41 Ti)
- 13.2-mL polypropylene thinwall tube (Beckman coulter, 331372)
- Refractometer (e.g., Carl Zeiss 120540)

1. **For EVs PKH26-labeling purification in sucrose density gradient ultracentrifugation (**S-DGUC**):**

- Lab-Tek II chamber Slide (ThermoFisher)
- Scanning confocal microscope (e.g., Leica TCS SP8, Mikrosysteme GmbH).

1. **Reagents**
2. **For sucrose density gradient ultracentrifugation (**S-DGUC**)**

- Phosphate buffer saline (PBS); pH 7.4
- Sucrose (e.g., Sigma-Aldrich)

| Stock solution (%) | 10 | 16 | 22 | 28 | 34 | 40 | 46 | 52 | 58 | 64 | 70 | 90 |
| --- | --- | --- | --- | --- | --- | --- | --- | --- | --- | --- | --- | --- |
| Sucrose (g) | 0.5 | 0.8 | 1.1 | 1.4 | 1.7 | 2.0 | 2.3 | 2.6 | 2.9 | 3.2 | 3.5 | 4.5 |
| Add PBS to 5 mL |  |  |  |  |  |  |  |  |  |  |  |  |

1. **For EVs PKH26-labeling purification**

- 100-200 µL of EVs
- PKH26 Red Fluorescent kit (Sigma-Aldrich)
- EVs-depleted fetal bovine serum (FBS; ThermoFisher)
- Bovine serum albumin (BSA)
- Paraformaldehyde (PFA) 4%
- Phalloidin (e.g., Atto 488, Sigma-Aldrich)
- ProLong™ Diamond Antifade Mountant with DAPI (ThermoFisher)

**3. Protocol**

1. **Purification of EVs in sucrose density gradient ultracentrifugation (S-DGUC)**

- Cool UC rotor to 4°C.
- Prepare sucrose stock concentrations 10-90% as a mentioned above.
- Determine sucrose density on a refractometer.
- Resuspend the EV pellets (100-200 μL) obtained in the final step of UC with 1 mL of 90% sucrose solution and transfer into 13.2 mL polypropylene tube.
- Mount eleven successive gradients with 1 mL of decreasing sucrose concentration starting with 70%. Technical tip: To apply sucrose solutions, touch the center (angled 90°) or side (angled 60°) of the ultracentrifuge tube with the end of 1000 μL pipet tip (cut approx.1cm) containing 1 mL sucrose solution and apply the solution carefully (S9A, B Fig). Avoid turbulence between each sucrose solution added. Note: thin borders or discs between each layer will be observed if layering was careful enough (S9C, D Fig).
- Balance the buckets (SW41 Ti) using 10% of sucrose solution if difference between buckets are noticed.
- Centrifuge samples for 16 h at 4°C for 200,000 x g without breaks. Approximately 3 h is the time the centrifuge takes to reach 0.
- Collect six fractions of 2 mL starting from the top to bottom. Carefully aspirate 1 mL with a 1000 μL pipet tip touching the top of solution and transfer it into another tube. Repeat this step again so that 2 mL fractions are collected.
- Add 9 mL of filtrated PBS (0.22µm) in each fraction, balance the buckets with PBS and centrifuge at 4 °C (130,000 x g for 1 hour) in a SW41 Ti rotor.
- Resuspend the final pellet in 50-100 µL of PBS and store at -80 °C for Western blotting or confocal microscopy assays.

**2. EVs PKH26-labeling in sucrose density gradient ultracentrifugation:**

- Resuspend the EV pellets (100-200 μL) obtained in the final step of UC with 400 µL of diluent C for 1 min (EVs+dC) into a 13.2 mL polypropylene tube. Separately, in a 1.5 mL microcentrifuge tube dilute 3 µL of dye PKH26 in 400 µL of diluent C (stain solution).
- Transfer the stain solution into 13.2 mL polypropylene tube content the EVs+dC and mix continuously by gentle pipetting. Let stand at room temperature for 3 minutes.
- Quench by adding 800 µL of 5% EVs-depleted FBS or 10% BSA in PBS for 2 min.
- Add 9 mL of fresh medium supplemented with 5% EVs-depleted FBS (referred to as EVs-medium), balance the buckets with medium and centrifuge at 4°C (130,000g for 1 hour) in a SW41 Ti rotor.
- Resuspend 100-200 µL of labeled EVs (referred to as PKH26-EVs) into a 13.2 mL polypropylene tube and repeat the **protocol 1** of sucrose gradient for UC, as a mentioned above. Note: Use labeled EVs as soon as possible to ensure highest possible fluorescent intensity.
- Add 30 µL of the pellet obtained from each fraction into macrophages (1-2 × 10^3^/per well, prepared as described in Material and Methods) culture on Lab-Tek II chamber slide and incubate at 37 °C for 3 h.
- After the internalization period, wash macrophages with PBS (twice). Fix the cells with 150 µL of 4% paraformaldehyde (PFA) for 20 min at room temperature. Wash for three time, then stain with 120 µL of Phalloidin-Atto 488 diluted 1:50 in PBS for 50 min and wash again for three time. Mount the slides with 2 or 3 drops of ProLong™ Diamond Antifade Mountant with DAPI and stored at 4 °C overnight. Finally, analyze the slides under a scanning confocal microscope.
