# Supplementary figures and images for: Characterization and internalization of small extracellular vesicles released by human primary macrophages derived from circulating monocytes
Source: PLoS One. 2020 Aug 24;15(8):e0237795. doi: 10.1371/journal.pone.0237795 (PMC7444811; doi:10.1371/journal.pone.0237795)

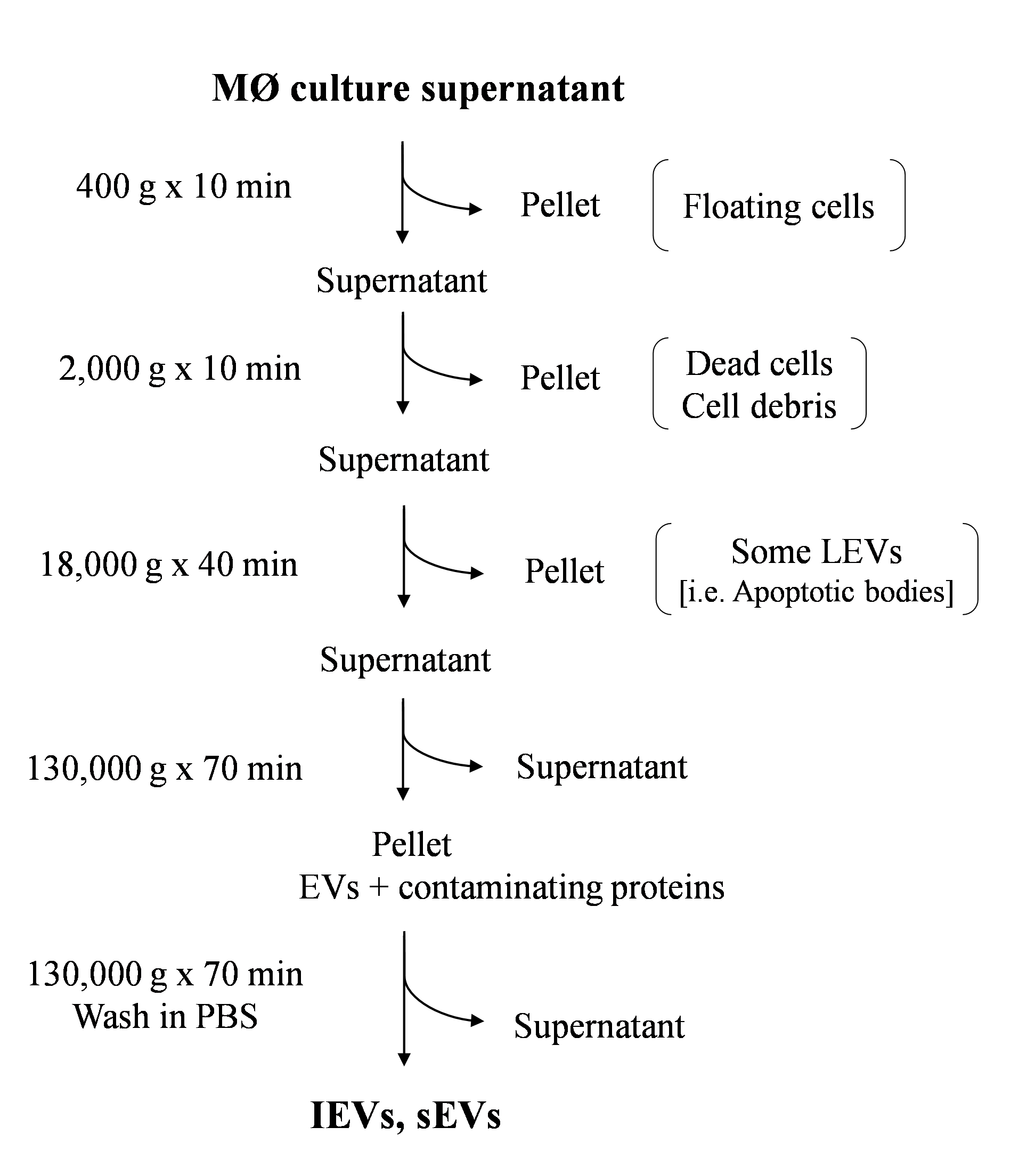

Supplement: S1 Fig — The flowchart shows the centrifugation steps applied for sEVs isolation from 10 mL of supernatants from monocytes-derived macrophages cultured in DMEM with 5% EVs-depleted serum. MØ: Macrophage; LEVs: large extracellular vesicles; IEVs: intermediated extracellular vesicles; sEVs: small extracellular vesicles. (TIF) [file pone.0237795.s002.tif]

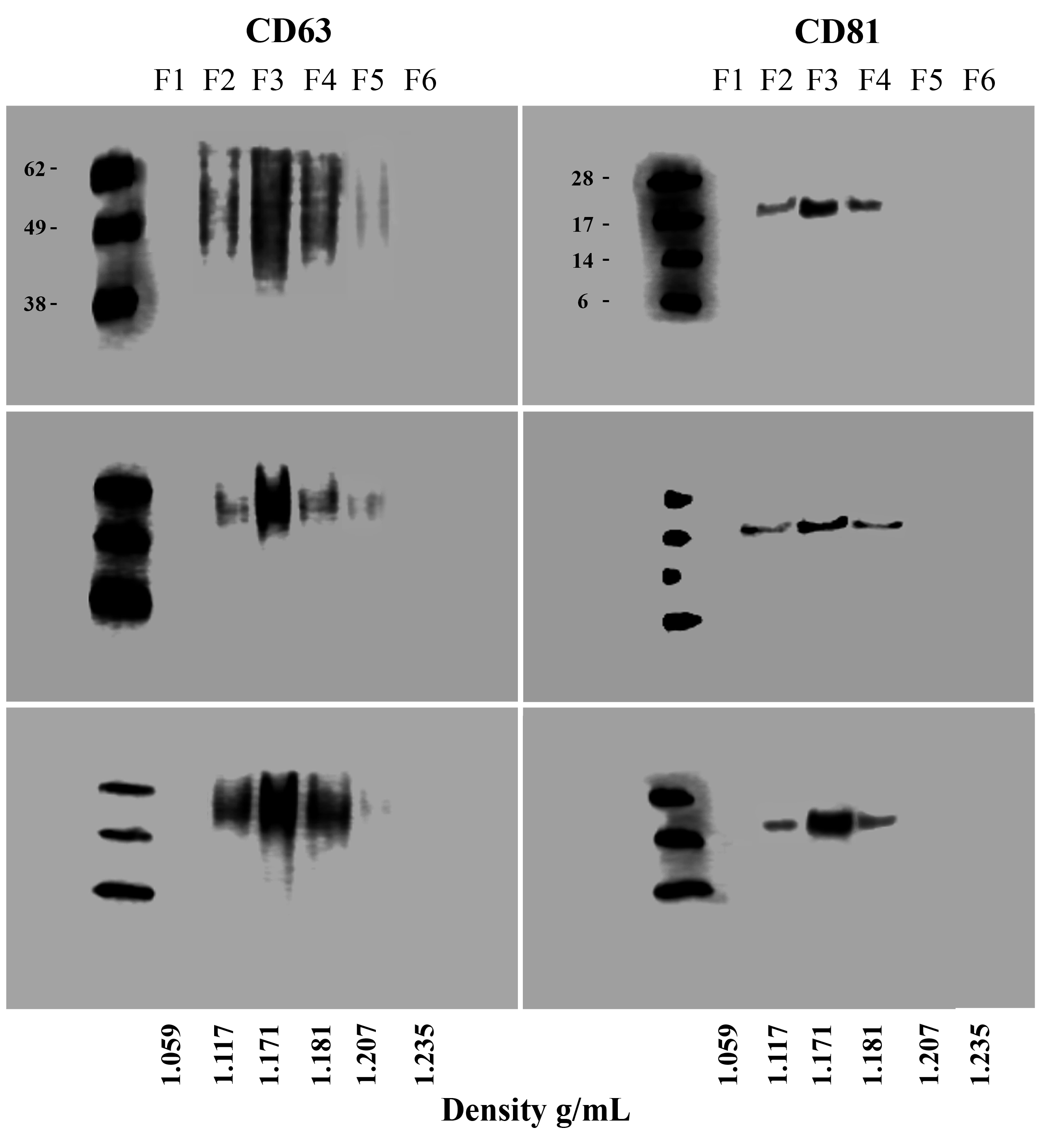

Supplement: S6 Fig — Polyacrylamide gels were loaded with 15 μg of protein and membranes were labeled with sEVs markers (CD63 and CD81). All experiments were performed with pools (4 donors) of sEVs. (TIF) [file pone.0237795.s007.tif]

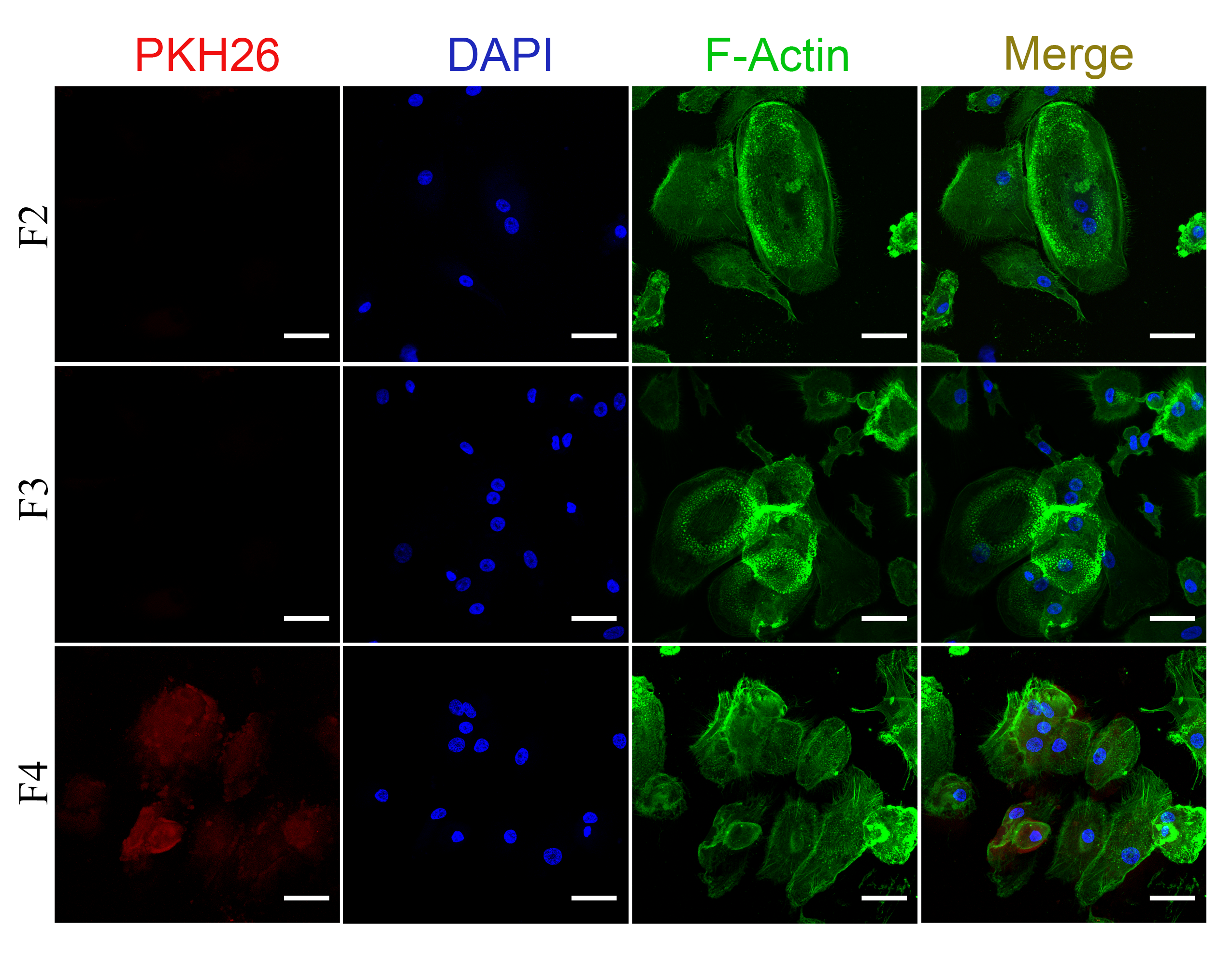

Supplement: S7 Fig — EVs-free medium were labeled with PKH26, then separated by sucrose density gradient centrifugation and three fractions (F2-F4) were colleted and added separately to recipient macrophages during 3 hours (n = 3). Fluorescent images represent only cells or cells with sEVs, respectively (Bars = 50 μm). PKH26: sEVs; DAPI: cell nuclei; F-Actin: macrophages. (TIF) [file pone.0237795.s008.tif]

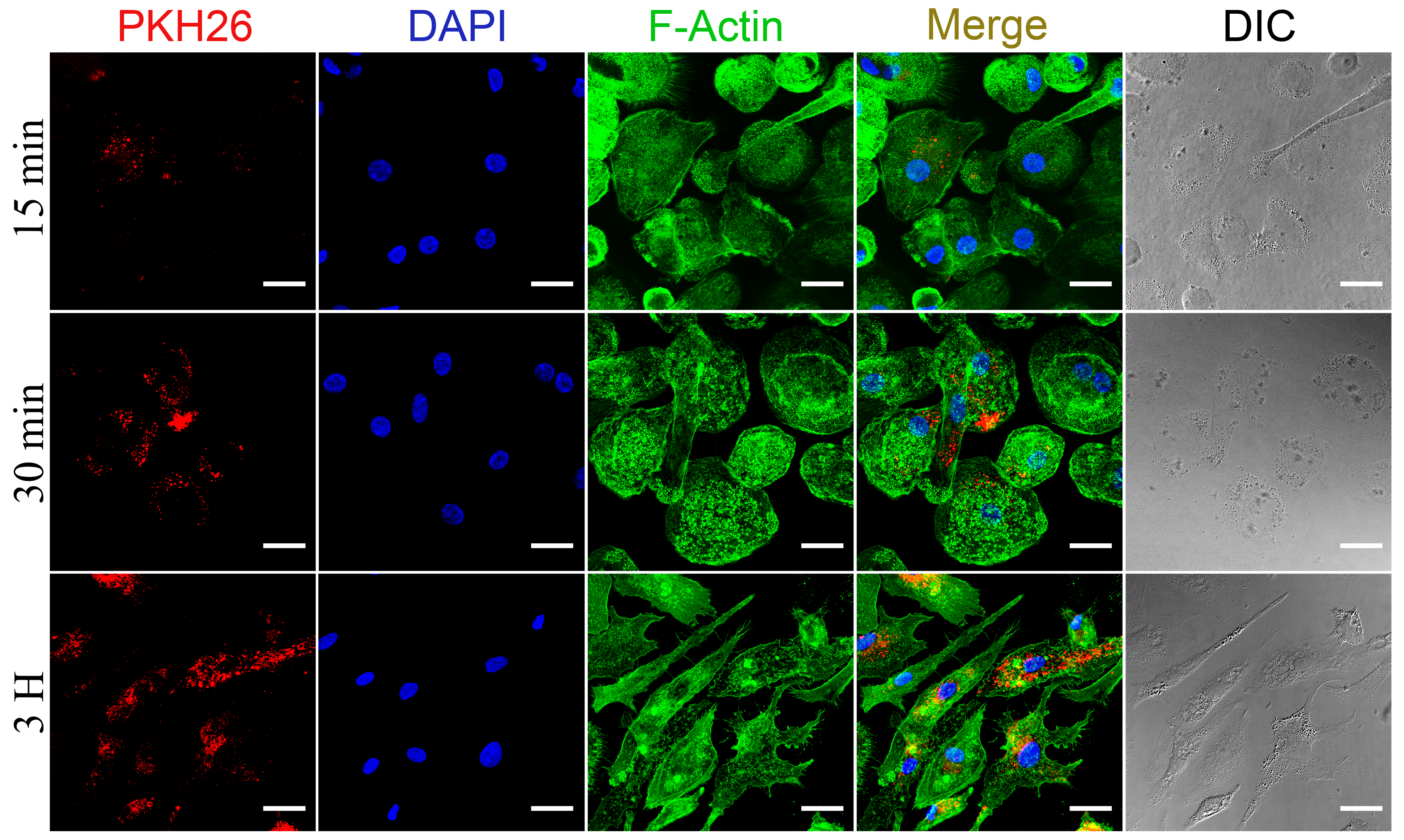

Supplement: S8 Fig — EVs pellets isolated by dUC (not purified by sucrose gradient) were labeled with PHK26 and added to macrophages during 15 min (n = 3), 30 min (n = 3), and 3 hours (n = 4). Bright field (DIC) and fluorescent images represent only cells or cells with extracellular particles, respectively (Bars = 50 μm). PKH26: EVs and other particles; DAPI: cell nuclei; F-Actin: macrophages. (TIF) [file pone.0237795.s009.tif]

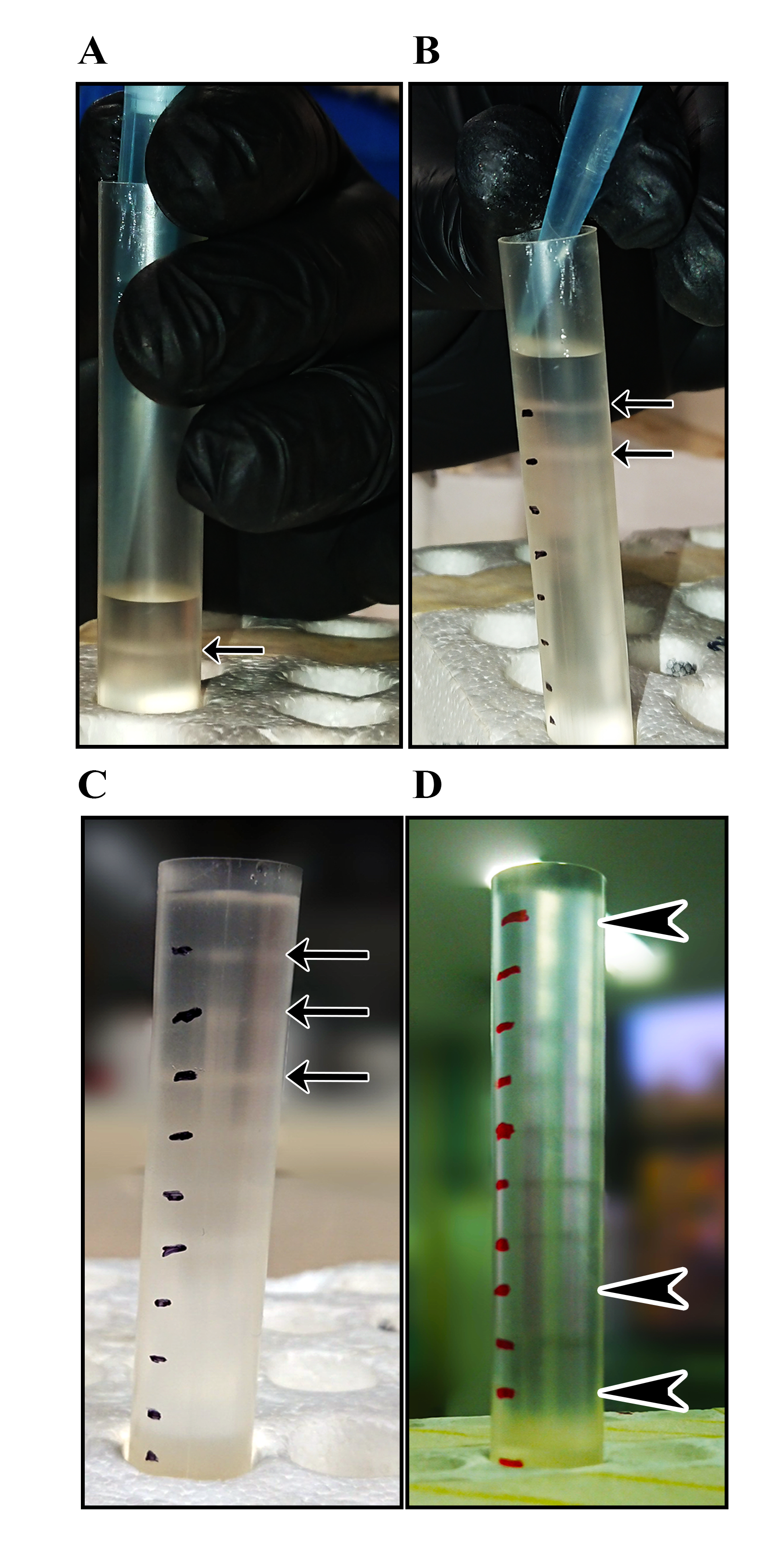

Supplement: S9 Fig — (A) Position of tube angled 90° and (B) angled 60° during layering sucrose gradients. (C) Correct and (D) incorrect sucrose gradients layers. Black arrows point to layer formation during sucrose gradient; black arrowheads point to diffuse layers. (TIF) [file pone.0237795.s010.tif]
