# Supplementary material for: Characterization and internalization of small extracellular vesicles released by human primary macrophages derived from circulating monocytes
Source: PLoS One. 2020 Aug 24;15(8):e0237795. doi: 10.1371/journal.pone.0237795 (PMC7444811; doi:10.1371/journal.pone.0237795)
Supplement: S1 Raw image — (PDF) [file pone.0237795.s011.pdf]

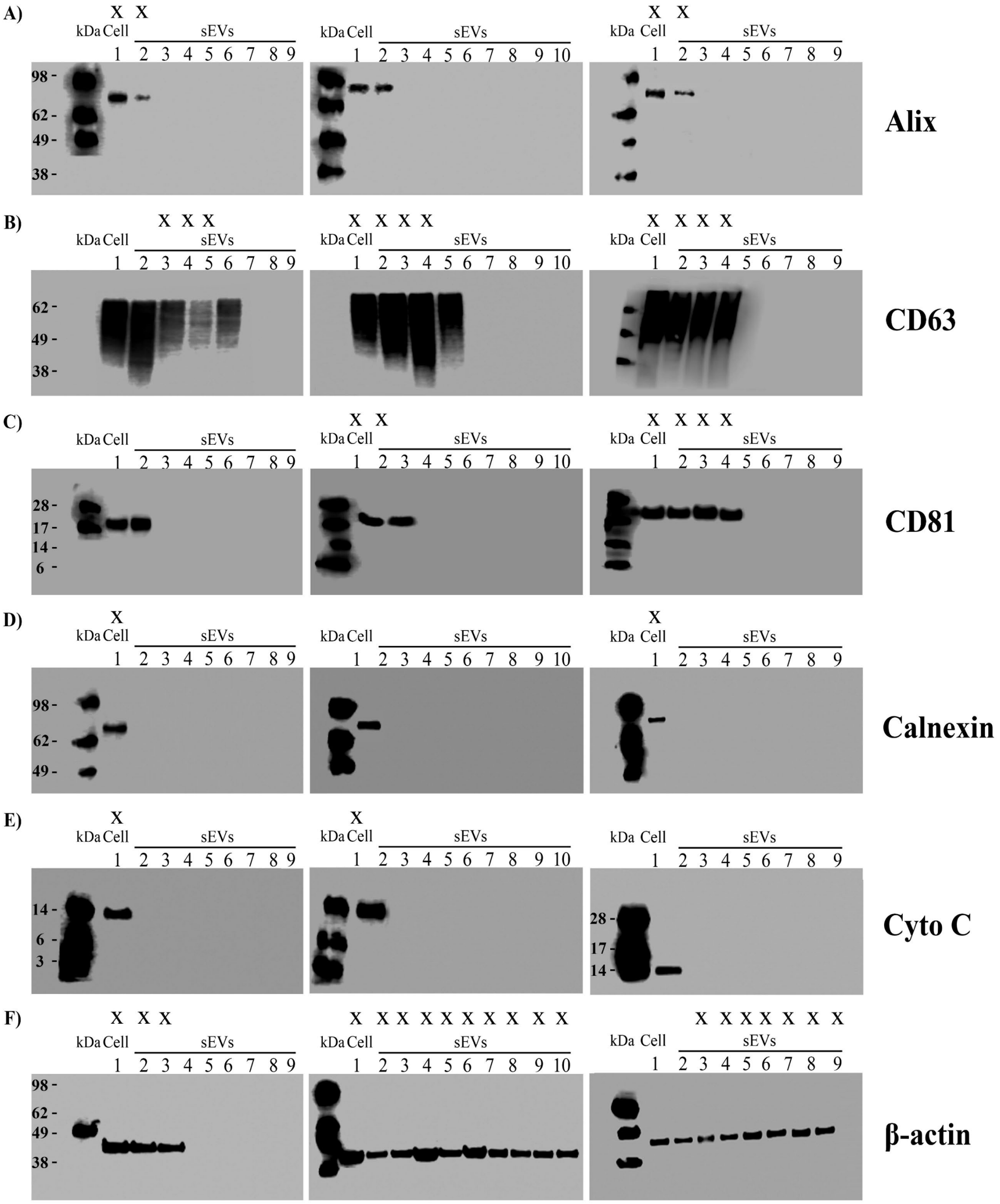

S5 Fig. Protein markers of MDM-derived sEVs. Original blots corresponding to Fig 4. Primary human macrophages (line 1) and pools of MDM-derived sEVs (lines 2-10). Fig 4 was generated from the following panels: Panel A (middle) for Alix; Panel B (left) for CD63; Panel C (left) for CD81; Panel D (middle) for Calnexin; Panel E (right) for Cyto C; Panel F (right) for β-actin. All blot images were captured using Image Studio Software (LI-COR imaging system). Lanes not included in Fig. 4 are marked with "X".

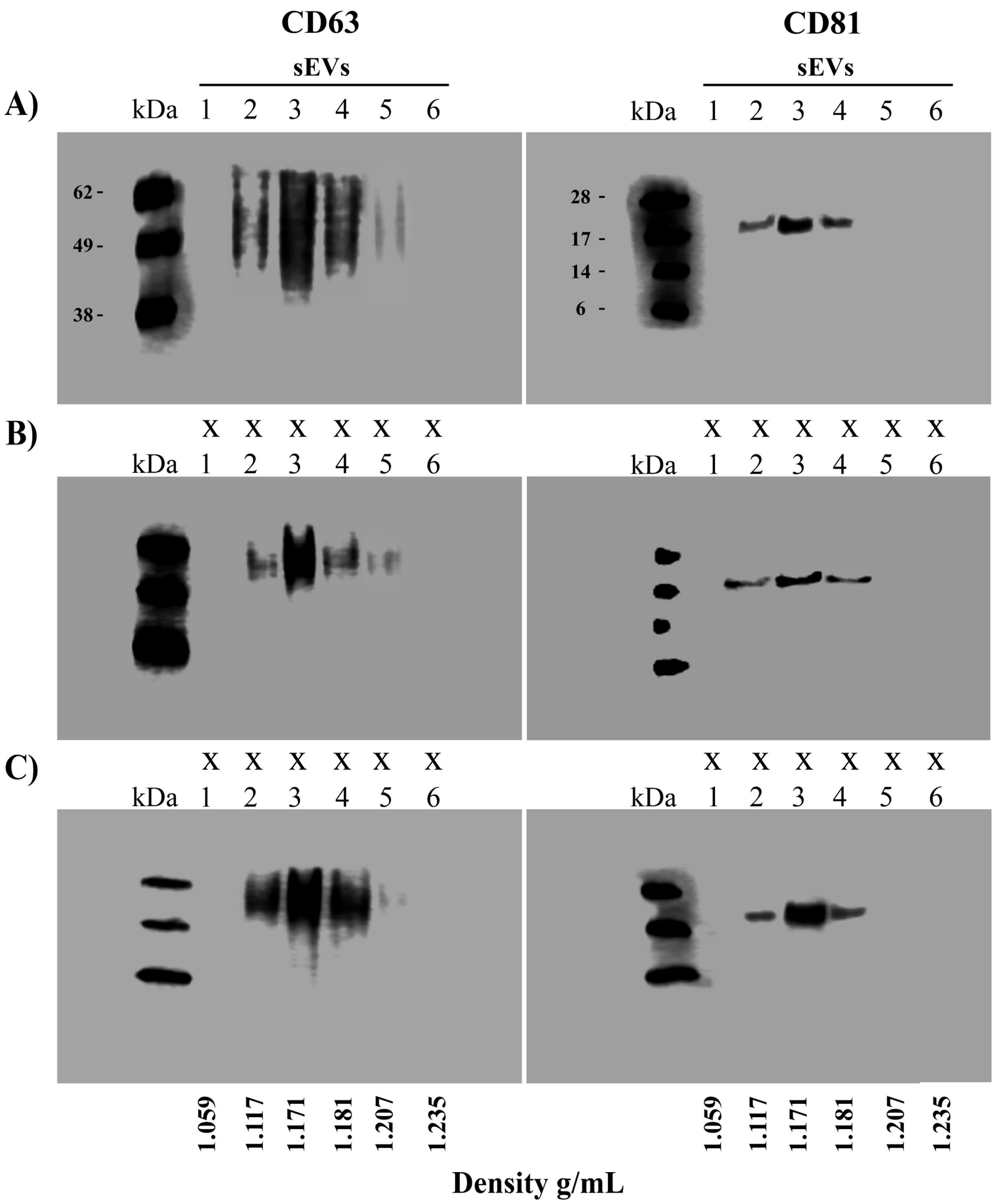

S6 Fig. Raw blot images of fractions F1-F6 collected of MDM-derived sEVs separated by S-DGUC. Original blots corresponding to Fig 5. MDM-derived sEVs (lines 1-6). Fig 5 was generated from panel A of CD63 and CD81 blots. All blot images were captured using Image Studio Software (LI-COR imaging system). Lanes not included in Fig. 5 are marked with "X".
